# Haemodialysis work environment contributors to job satisfaction and stress: a sequential mixed methods study

**DOI:** 10.1186/s12912-015-0110-x

**Published:** 2015-11-10

**Authors:** Bronwyn Hayes, Ann Bonner, Clint Douglas

**Affiliations:** 1 Haemodialysis Unit, Cairns Hospital, c/o Renal Unit, P.O. Box 902, Cairns, QLD Australia; 2 School of Nursing, Queensland University of Technology, Brisbane, Australia; 3 Kidney Health Service, Metro North Hospital and Health Service, Brisbane, Australia

**Keywords:** Job satisfaction, Job stress, Burnout, Work environment, Mixed-methods, Renal, Haemodialysis

## Abstract

**Background:**

Haemodialysis nurses form long term relationships with patients in a technologically complex work environment. Previous studies have highlighted that haemodialysis nurses face stressors related to the nature of their work and also their work environments leading to reported high levels of burnout. Using Kanters (1997) Structural Empowerment Theory as a guiding framework, the aim of this study was to explore the factors contributing to satisfaction with the work environment, job satisfaction, job stress and burnout in haemodialysis nurses.

**Methods:**

Using a sequential mixed-methods design, the first phase involved an on-line survey comprising demographic and work characteristics, Brisbane Practice Environment Measure (B-PEM), Index of Work Satisfaction (IWS), Nursing Stress Scale (NSS) and the Maslach Burnout Inventory (MBI). The second phase involved conducting eight semi-structured interviews with data thematically analyzed.

**Results:**

From the 417 nurses surveyed the majority were female (90.9 %), aged over 41 years of age (74.3 %), and 47.4 % had worked in haemodialysis for more than 10 years. Overall the work environment was perceived positively and there was a moderate level of job satisfaction. However levels of stress and emotional exhaustion (burnout) were high. Two themes, *ability to care* and *feeling successful as a nurse*, provided clarity to the level of job satisfaction found in phase 1. While two further themes, *patients as quasi-family* and *intense working teams*, explained why working as a haemodialysis nurse was both satisfying and stressful.

**Conclusions:**

Nurse managers can use these results to identify issues being experienced by haemodialysis nurses working in the unit they are supervising.

## Introduction

Haemodialysis nurses provide care to patients with end stage kidney disease (ESKD) who require renal replacement therapy. Haemodialysis provides ongoing life sustaining treatment until the patient receives a renal transplant or dies [1]. Patients in Australia and New Zealand most commonly receive dialysis three times per week for 4–5 h on each occasion [2] in a variety of settings (hospitals, free-standing units, homes; more comprehensively described in Agar et al., 2007). The total time required to prepare, deliver and discontinue a haemodialysis treatment is approximately 6 h. Therefore haemodialysis nurses frequently care for the same patient up to three times a week for an extended period of time, often years and in some cases decades, leading to unique nurse-patient relationships [3–5].

The haemodialysis work environment is highly technical [6] with nurses needing to master complex haemodialysis equipment to provide safe, efficient and effective care to patients. Haemodialysis nurses are required to fulfil many demanding roles such as advocate, caregiver, educator, mentor and technician while patients attend a dialysis unit [7]. The complexities of the role that are performed by these nurses along with organizational factors within the work environment have led to haemodialysis nurses experiencing high levels of burnout. For instance in the US 1 in 3 experienced burnout [8], 52 % in Australia and New Zealand [9]. Kavurmaci et al. [10] found medium to high levels of emotional burnout in haemodialysis nurses in a small Turkish study. High levels of nurse burnout contribute to poor patient outcomes, increased sick leave, decreased organizational commitment and increases in staff leaving their work environment and even the profession of nursing [11, 12]. Where the work environment is viewed favorably by nurses, there are higher levels of job satisfaction [13], organizational commitment, retention of staff [14] and improved patient outcomes [15, 16]. The purpose of this study is to gain an understanding of the factors that contribute to job satisfaction, stress and subsequent burnout in haemodialysis nurses. Results can assist nurses and nurse managers to identify causative factors which could lead to greater job satisfaction, organizational commitment and retention of specialist haemodialysis nurses and improved patient outcomes.

## Background

Previous research has demonstrated that empowering and supportive work environments improve levels of job satisfaction and decrease job stress, and the incidence of burnout in nurses [17–20]. The work environment refers to the physical-social-psychological characteristics of the work setting [21]. A professional work environment encourages nurses to have control over the delivery of patient care and the environment where the care is delivered [20]. In a large multinational study involving over 1400 hospitals across nine countries, Aiken et al., [17] found that between 25-33 % of hospitals had poor work environments.

The guiding framework for this study was Kanter’s Structural Empowerment Theory which examines power and structural factors within the work environment and the resultant influence on job satisfaction, stress and burnout [22, 23]. Power and structural factors are known to influence the work environment [23]. Kanter [22] claims that power can originate through both formal and informal pathways and it influences the ability to access the empowering work structures. Formal power is derived from the position that the employee has in the organisation and the characteristics of the job. Informal power also exists and is more subtle than formal power; it develops from contact with colleagues and social alliances which arise both inside and outside of the organisation [24]. Empowering work structure includes access to: information (the ability to be involved with organizational decisions, policies and goals and pass information on to other colleagues); support (receiving feedback and guidance from supervisors, peers and subordinates to enable employees to have the ability to take action in response to situations with difficulty); resources (access to money, materials, supplies and equipment required to achieve organizational goals); and opportunities (access to professional development opportunities to increase knowledge and skills in order to advance within the organization) [22, 23]. O’Brien [25] found that when structural factors were missing or reduced, hemodialysis nurses reported higher levels of burnout. According to Kanter [22, 23] access to these factors has a greater impact on work attitudes and behaviors than the personality traits that an employee may possess. Previous research has found that the hemodialysis work environment is stressful [26] and intense [27]. With the tightening of health budgets, the nursing profession has been affected with job losses, pay cuts, decreased working conditions, replacement of nurses with unregulated, lower educated healthcare assistants, increased workloads, and increased stress [28]. These environmental factors affect the level of job satisfaction that a nurse can achieve [29].

Job satisfaction is “how employees actually feel about themselves as workers, their work, their managers, their work environment, and their overall work life” ([29] p. 132). Job satisfaction is multifaceted and complex due to the interaction between intrapersonal, interpersonal and extra-personal factors that contribute to overall job satisfaction [30, 31]. Literature highlights the positive effect that job satisfaction can have on the retention of nurses. Psychological engagement [32], enhanced organizational commitment [14] and morale [33] are by-products of high job satisfaction levels [34]. High levels of nurse job satisfaction have also been associated with improved positive outcomes for patients with decreased adverse events [35]. Conversely, lower levels of job satisfaction are due to high workloads (nurse-to-patient ratios), dissatisfaction with pay, poor communication with managers, and a lack of clinical autonomy [36]. Low levels of job satisfaction have been associated with increased patient morbidity and mortality [15], nurse burnout [11] and increased nurse turnover leading to nursing workforce shortages [37, 38].

The work environment and nurses’ demographic profiles have been previously identified as contributing to the level of haemodialysis nurses’ job satisfaction [9, 39]. The type of haemodialysis setting contributes to job satisfaction with nurses working in in-center acute units demonstrating less job satisfaction than those who worked in a home haemodialysis unit where the practice has greater autonomy and patients are more independent [9]. Older nurses and those who have worked in the haemodialysis environment longer are also known to have higher levels of job satisfaction [9, 40] while those that have less than three years’ experience have been identified as having the lowest levels of job satisfaction [9]. Other factors contributing to greater levels of job satisfaction in haemodialysis nurses include having time to meet the psychological and physical needs of patients, and the delivery of quality patient care [27, 41].

According to Lazarus and Folkman [42], job stress is ‘a particular relationship between the person and the environment that is appraised by the person as taxing or exceeding his or her resources and endangering his or her well-being p.19’. Higher levels of stress contribute to lower job satisfaction [18], poorer patient outcomes [15], increased burnout [43] and higher turnover of nursing staff [37]. Stress can also become persistent (or chronic) which can result in burnout that is characterized by decreased personal accomplishment, depersonalization and emotional exhaustion [44]. Job stress and burnout in haemodialysis nurses has been attributed to higher workloads [45, 46], poor interpersonal relationships with colleagues [4, 40, 47], ineffective communication with management [47, 48], intense patient-nurse relationship [49], violence and aggression from patients [48], and discrimination directed at nurses from patients.

Increasingly the work environment that haemodialysis nurses work in has been under multi-faceted pressure [28]. In the context of recent fiscal pressures in healthcare [50], a rapidly rising global burden of chronic kidney disease and more patients requiring haemodialysis [51], and an increasing recognition that haemodialysis nursing is stressful, there is a need to explore, for the first time, the work environment in which these nurses work and the resultant impact on job satisfaction, stress and burnout.

This study sought to answer the following research questions:

1. What is the level of satisfaction with the work environment, overall job satisfaction, stress and burnout for nurses working in the hemodialysis setting?
2. What are the relationships among the work environment, job satisfaction, job stress and burnout?
3. How do hemodialysis nurses understand the nature of their nursing work in relation to job satisfaction, job stress and burnout?

## Methods

### Design

**Fig. 1:**
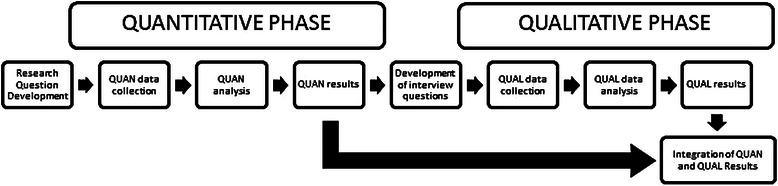
Research Process

A sequential explanatory mixed methods design [52] was used, beginning with a cross-sectional online survey, followed by individual semi-structured interviews. Using this explanatory design, specific quantitative findings that warrant further investigation such as unexpected results or unexplained differences are identified and then clarified through qualitative methods [53]. The two phases are connected at the beginning of the study with the development of both quantitative and qualitative research questions, between the quantitative and qualitative phases while developing questions for qualitative interviews. Integration of results occurred following quantitative and qualitative data analysis. In this study, the quantitative phase was dominant with the qualitative phase taking a secondary explanatory role (see Fig. 1). This method was chosen as the factors to be explored had previously been identified in the literature and pre-existing validated measures were available to explore the main study variables. The use of a sequential explanatory design also provided a structured approach to the research process.

### Participants

A cross-sectional sample of haemodialysis nurses was drawn from the Renal Society of Australasia (RSA) membership. The RSA is the peak body for nurses and dialysis technicians providing renal care in Australia and New Zealand with approximately 1328 members (April 2011). These members may be working in a variety of roles within renal care such as haemodialysis, peritoneal dialysis, chronic kidney disease, education or transplantation. Only registered (completed the required education preparation typically a 3 year Bachelor degree) and enrolled nurses (completed a one to two year training course, works under the supervision of a registered nurse) working more than five shifts per fortnight in the haemodialysis environment were invited to participate in the quantitative phase. At the conclusion of the quantitative data collection, participants indicated their willingness to participate in the qualitative phase of the project.

### Data collection

The quantitative phase consisted of an online survey that included demographic and work characteristics questions, and measures of the work environment, job satisfaction, job stress and burnout. Data were collected between October 2011 and April 2012.

The Brisbane Practice Environment Measure (B-PEM) is a 26 item measure examining four sub-components of the work environment: getting things done, professional development, flexibility in management support and feeling valued [54]. A high score denotes satisfaction with the work environment.

The Index of Work Satisfaction (IWS) is a commonly used to measure six components of nurse job satisfaction: *pay* (the dollar remuneration or fringe benefits received for work done), *autonomy* (the amount of job related independence, initiative and freedom, either permitted or required, in daily work activities), *task requirements* (tasks or activities that must be done as a regular part of the job), *organisational policies* (management policies and procedures put forward by the hospital and nursing administration of the hospital), *interaction* (opportunities presented for both formal and informal social and professional contact during working hours) and *professional status* (overall importance or significance felt about your job, both in your view and in the view of others) [55, 56]. A high score out of 7 for the sub scales suggests high job satisfaction.

The Nursing Stress Scale (NSS) consists of 34 items presented in 7 sub scales that describe situations that have been identified as causing stress for nurses in the performance of their duties [57]. The sub components include: death and dying, conflict with physicians, inadequate preparation, lack of support, conflict with other nurses, workload, and uncertainty regarding treatment. Scores for the sub scales of the NSS ranged from 1–4 with a high score suggesting high levels of stress.

The Maslach Burnout Inventory (MBI) measures employees’ feelings about work and is centred around the three identified aspects of burnout: *emotional exhaustion* – the feeling of being emotionally overextended and exhausted by one's work, *depersonalisation* – the unfeeling and impersonal response towards recipients of one's service, and *personal accomplishment* – the feeling of competence and successful achievement in one's work [58]. The emotional exhaustion sub scale is described as the core measure of burnout [44]. High levels of burnout are indicated when emotional exhaustion scores are above 28, depersonalisation scores are above 10 and personal accomplishment scores are above 40 [59].

All instruments demonstrated reasonable to good model fit using standard fit indices. Internal reliability of each measure was also examined using Cronbach’s alpha. The B-PEM sub scales ranged from .80-.86, with an overall alpha of .91. The IWS (Part B) sub scales ranged from .72-.85, with an overall alpha of .90. The NSS sub scales ranged from .70 to .85, with an overall alpha of .82. Finally, the emotional exhaustion scale of the MBI demonstrated good internal reliability, with an alpha of .92 [39].

The qualitative phase consisted of semi-structured interviews that were conducted between May 2013 and July 2013. In total fifty participants had expressed a willingness to be involved in the qualitative phase. Progressively groups of 10 were contacted by email and invited to participate in the qualitative phase. The email contained an information sheet and consent form to be returned if they were willing. Once those participants had responded a further group of 10 was selected to participate. This procedure continued to select participants using a maximum variation sampling procedure [60] to ensure that characteristics identified as variables of interest in the quantitative phase (gender, type of dialysis unit [in-center, satellite and home therapies], work location [metropolitan, regional, rural and remote] and length of time working in haemodialysis) were included. Interview questions were formulated following analysis of the quantitative data with the aim to investigate the study variables more fully and to explore unexpected results (see Table 1). Interviews were 40–60 min duration, digitally recorded and transcribed verbatim for analysis.

**Table 1:** Interview Questions

INTERVIEW SCHEDULE
Preliminary/introduction
•Can you tell me how long you have been working as a haemodialysis nurse and why you have come to be working in haemodialysis?
*Satisfaction*
•What is it about being a haemodialysis nurse that gives you the most satisfaction? Why?
•Think back over your time in haemodialysis, what has satisfied you the most?
•What is it that you actually do during a shift that gives you satisfaction?
•What do you find least satisfying about your job? Why?
*Work Environment*
•How does the work environment contribute to job satisfaction
•Does regular, ongoing contact with patients contribute to satisfaction or is it stressful? Why?
•How does the dialysis work environment contribute to job stress for you?
*Stress*
•Can you tell me about stressors that you experience in the haemodialysis unit?
•Have you found it more or less stressful the longer you have stayed working as a haemodialysis nurse? Why?
•I found in the first phase of this research that coping with death and dying was a stressor, how do you cope when a patient deteriorates and dies?
•During the first phase we found that haemodialysis nurses had a high level of burnout? Why do you think this would be the case from your perspective?
*Burnout*
•During the first phase we found that nurses working in in-center dialysis units had higher levels of burnout compared to those who work in satellite and home haemodialysis, why do you think this might be from your point of view?
•Have you come close to resigning or leaving the haemodialysis unit? Why have you decided to stay or go?
*General*
•What do you believe would improve your workplace for haemodialysis nurses?

### Ethics

All study procedures were approved by the University’s Human Research Ethics Committee prior to commencing data collection. At the beginning of each phase detailed study information was provided to potential participants. Completion of the online survey implied consent, whereas written consent was obtained prior to interviews being conducted.

#### Data analysis

The two data sets were analyzed separately. At the conclusion of the analysis the quantitative and qualitative results were integrated to explain how haemodialysis nurses viewed their work environment in relation to job satisfaction, stress and burnout.

### Quantitative

Quantitative data analysis was performed using IBM SPSS Statistics version 21 software. In this study there was a “drop-out” of participants as they progressed through the online survey (92 % completed the B-PEM, 82 % the IWS, 79 % the NSS, with an overall item completion of 78 %). Data was assessed using Little’s MCAR as missing completely at random, and the best option for replacing missing data was through multiple imputations [60]. Descriptive statistics were used to summarize the sample characteristics. ANOVA and *t*-tests were used to compare the components of the work environment, job satisfaction, job stress and burnout measures with nurse and work characteristics. Pearson’s correlations coefficients were also used to explore the relationships between the main study variables. Finally, multivariable modeling using structural equation modelling was conducted to test an explanatory model of the relationships between the work environment, job satisfaction, job stress and emotional exhaustion based on the theoretical framework. Statistical significance was *p* < 0.05 for all analyses.

### Qualitative

Interview transcripts were thematically analyzed following the steps described by Braun and Clarke [61]: (1) becoming familiar with the data, (2) generating initial codes, (3) searching for themes, (4) reviewing themes, (5) defining and naming themes, and (6) producing the report. Significant statements were extracted and meanings were formulated into themes. Steps 3–5 were undertaken by the authors (BH and AB) independently and then together to clarify emerging themes. At this point, individual transcripts were checked for the presence of each theme, and it was agreed that data saturation had been reached. Braun and Clarke’s analytical steps also assisted with demonstrating rigor through the development of conceptualized and rich themes.

### Integration

Integration is the “relationship between two or more methods where the different methods retain their paradigmatic nature but are inter-meshed with each other in pursuit of the goal of “knowing more p.51” [62]. In this study integration occurred after the emergence of the qualitative themes and involved the qualitative themes providing an explanatory role to the dominant quantitative results.

### Rigor

In an explanatory mixed methods design, minimizing threats to validity are needed. Strategies used in this study were: using a large sample and reliable instruments during the dominant quantitative phase; choosing significant quantitative results to follow up on in the qualitative phase; selecting the same individuals for the qualitative follow-up from the quantitative phase; and analysing the qualitative data independently and then comparatively [63]. Finally credibility and transferability comes from the overall inferences of the integrated results rather than from the qualitative results themselves [64].

## Results

Following a sequential explanatory design [65, 66], results for the dominant quantitative phase will be presented first followed by the qualitative results that will build on and seek to provide greater understanding and explanation of the quantitative results.

### Participant characteristics

417 nurses completed the online survey and a summary of the sample demographics are presented in Table 2. A majority of nurses were female (90.9 %), aged over 41 years of age (74.3 %), and 47.4 % had worked in haemodialysis for more than 10 years. For the qualitative phase, eight nurses (6 female) were interviewed who worked in various locations and types of haemodialysis units (see Table 3). The length of time working in this field of nursing varied from 1 year to over 20 years.

**Table 2:** Sample Demographics

		Number	Percent
Gender	Female	379	90.9
	Male	38	9.1
Country	Australia	396	94.9
	New Zealand	21	5.1
Age (years)	21-30	23	5.5
	31-40	84	20.1
	41-50	156	37.4
	51-60	141	33.8
	60+	13	3.1
Length of Time Working in Haemodialysis	<1 year	11	2.6
	1-2 years	22	5.3
	3-5 years	70	16.8
	6-10 years	116	27.8
	11-15 years	94	22.5
	16-20 years	41	9.8
	>20 years	63	15.1
Nursing Classification	Registered Nurse (RN)	406	97.4
	Enrolled Nurse (EN)	11	2.6
Highest Nursing Qualification	Certificate in Nursing	85	20.4
	Diploma in Nursing	56	13.4
	Undergraduate Degree	74	17.7
	Postgraduate Certificate/Diploma	170	40.8
	Masters/Doctorate	32	7.7
Work Location	Metropolitan	177	42.4
	Regional	144	34.5
	Rural	85	20.4
	Remote	11	2.6
Type of Unit	In-Center	187	44.8
	Satellite	202	48.4
	Home	28	6.7

**Table 3:** Characteristics of participants (Qualitative Phase)

Characteristics	Participants							
	1	2	3	4	5	6	7	8
Gender								
Female	✓	✓	✓		✓	✓	✓	
Male				✓				✓
Type of Dialysis Unit								
In-center				✓		✓		✓
Satellite		✓	✓				✓	
Home therapies	✓				✓			
Work Location								
Metropolitan	✓			✓				
Regional		✓			✓			✓
Rural			✓					
Remote						✓	✓	
Length of time working in haemodialysis								
1-2 years			✓					
3-5 years	✓					✓		✓
6-10 years				✓				
11-15 years		✓					✓	
16-20 years					✓			✓

### Quantitative phase

Descriptive results (Table 4) reveal that flexible management (M = 3.74, *SD* = 0.75) and feeling valued (M = 3.65, *SD* = 0.68) were factors contributing to the most satisfaction with the work environment. Nurses who had worked in haemodialysis the longest (> 20 years) had the highest overall mean job satisfaction score (M = 3.60, *SD* = 0.69) while nurses who had worked the shortest time (<1 year) had lower satisfaction scores (M = 3.12, *SD =* 0.54) and higher overall levels of stress (M = 74.55, *SD =* 13.85). Greatest job satisfaction was derived from their professional status (*M* = 5.35, *SD* = 0.89), followed by informal and formal interactions in the workplace (*M* = 4.88, *SD* = 1.05) and autonomy (*M* = 4.84, *SD* = 1.08). Using the Nursing Stress Scale, the highest stressors were workloads (M = 2.29, *SD* = 0.52) and death and dying (M = 2.19, *SD* = 0.47). Conflict with nurses scored the lowest across the stress subscales (M = 1.95, *SD* = 0.51). Across the sample, high levels of burnout were found with 52.5 % of nurses reporting high levels of emotional exhaustion even though high levels of job satisfaction and satisfaction with the work environment were present. The work environment was found to be highly correlated with job satisfaction (*r* = 0.70, *p* < 0.01). Lower job satisfaction was associated with higher levels of stress (*r* = −0.52, *p* < 0.01) and emotional exhaustion (*r* = −0.56, *p* < 0.01).

**Table 4:** Summary Statistics for Haemodialysis Nurses’ Work Environment, Job Satisfaction, Stress and Burnout

				Range	
	Sub-scales & Total	*M*	*SD*	Potential	Actual
Work Environment (B-PEM)	Getting Things Done	3.56	0.60	1-5	1-5
	Flexible Management Support	3.74	0.75	1-5	1-5
	Feeling Valued	3.65	0.68	1-5	1-5
	Professional Development	3.30	0.80	1-5	1-5
	Total B-PEM score	92.46	15.02	26-130	30-124
Job Satisfaction (IWS)	Pay	3.53	1.11	1-7	1.00-6.67
	Professional Status	5.35	0.89	1-7	2.71-7.00
	Interactions	4.88	1.05	1-7	1.90-7.00
	Autonomy	4.84	1.08	1-7	1.57-7.00
	Task Requirements	4.05	1.04	1-7	1.33-7.00
	Organizational Policies	3.68	1.11	1-7	1.00-6.57
	Total IWS score	191.16	31.19	44-308	98-276
Job Stress (NSS)	Death and Dying	2.19	0.47	1-4	1.00-3.71
	Conflict with Physicians	2.04	0.45	1-4	1.00-3.60
	Inadequate Preparation	2.07	0.50	1-4	1.00-3.67
	Lack of Support	1.98	0.63	1-4	1.00-4.00
	Conflict With Other Nurses	1.95	0.51	1-4	1.00-3.80
	Workload	2.29	0.52	1-4	1.00-4.00
	Uncertainty Concerning Treatment	2.04	0.50	1-4	1.00-3.80
	Total NSS score	71.48	12.16	34-136	34-105
Burnout (MBI)	Emotional Exhaustion	29.59	12.11	9-63	9-63
	Personal Accomplishment	39.93	7.29	5-56	8-56
	Depersonalization	11.89	6.51	5-34	5-34
	Total score (not applicable)				

Multivariable modeling using structural equation modelling identified that the work environment had a direct positive effect on job satisfaction (β = 0.94, *p* < .01), greater job satisfaction predicted lower stress (β *= −*0.91, *p* < .05), job satisfaction had an indirect effect on emotional exhaustion through job stress (β = −0.59) and job satisfaction did not have a direct effect on emotional exhaustion (β = 0.07, *p* = 0.82). The work environment accounted for 88 % of the variance in nurses’ job satisfaction. Job satisfaction explained 82 % of the variance in job stress. Job satisfaction and job stress together explained 34 % of the variance in emotional exhaustion scores. Further in-depth reporting of the descriptive results, correlations and the structural equation model have been published elsewhere [9].

### Qualitative phase

Four explanatory themes emerged from the qualitative data. These were: ability to care, patients as quasi-family, feeling successful as a nurse, and intense working teams.

#### Ability to care

The ability to care theme captures nurses’ descriptions of being able to give “good” care and having the time to care. The ability to provide holistic, respectful, patient-centered care along with a level of empathy was highlighted as the qualities required to provide good care to patients receiving haemodialysis. The ability to provide “good” care was spoken about in relation to both job satisfaction and the work environment. This theme encompassed having time to assess patients, complete care plans and being able to spend time with patients experiencing personal difficulties. When the ability to care was compromised by excessive workloads, care became task oriented with nurses unable to meet the psychological needs of the patients and job satisfaction was compromised. For instance one nurse stated that:

*It's more than shoving needles in and putting people on and coming back and taking the needles out and sending them home. That doesn’t give me that much satisfaction. What gives me the satisfaction is being able to talk to them and work out where I can better help them achieve a better standard of living. (George)*

#### Patients as quasi-family

Nurses described that close relationships were formed with patients and this contributed to both job satisfaction and stress; a contradiction to the quantitative results which emerged during the qualitative phase. The nurses identified that the nature of haemodialysis nursing led to a “special relationship” to form with patients due to the regular, ongoing and prolonged interaction that occurs in this work setting. The relationships form over many years (occasionally decades) because of the repeated contact with the same patient. Nurses described how the relationship led to increased familiarity, “closeness” and blurring of traditional therapeutic relationships. One nurse stated that:

*I really tried to do the boundary settings as well as possible. But it was like hanging around with, yeah, a bunch of fun uncles and aunties that sort of care about you and look out for you and treat you like a family member. (Fred)*

This intense prolonged interaction led to a ‘quasi-family like’ relationship forming between the nurse and ‘their’ patients. While this long-term continuity of care was seen as a source of satisfaction it also meant that nurses often became involved in family matters affecting the patient. Nurses reported giving counsel for family difficulties, divorces, being invited to weddings, birthday parties and funerals of patients’ family members. This closeness was particularly problematic when patients died. Nurses described grief, sometimes unresolved grief at the death of a patient that they had come to know. For instance:

*I think it* [close contact with patients] *can be stressful in that obviously you get more attached to some patients than others and one of our patients who we pretty much had daily contact with, he just used to ring up and have a bit of a chat with for 5–10 min. He was 42. He was a very very sick man and he died last Easter and we are all still grieving for him (Susan)*

The close connection with the patient, while for the most part provided satisfaction in the form of being able to provide psychological support for the patient also led to an emotional burden for the nurse. Nurses described how this relationship could potentially lead to burnout particularly when a patient died and there was inadequate support from the organization for the nurse to process the grief and loss.

*You know, I had a patient die a month ago, I wasn’t told by the hospital that someone that I’ve known for five years, who I’ve bonded with who, just someone that you get close to and then they die, and you don’t get the counselling, you don’t get the support, you don’t get the resolution of grief because it’s strongly recommended that you don’t go to funerals. It’s really bizarre and I don’t really know how to cope with that. Especially since I’m not really an emotional person. (Mary)*

#### Feeling successful as a haemodialysis nurse

Nurses reported that the job itself was rewarding largely due to the duties performed that were satisfying, and gave a sense of achievement. The nurses described the positive feeling which revolved around being technically proficient, having autonomy in making practice decisions and overall feelings of success. Proficiency and a sense of pride in the complex and challenging technical aspects of safely performing haemodialysis treatment were highlighted by these nurses as a way to explain their satisfaction. It was the underlying intrinsic reward (sense of achievement) derived from being able to provide treatment that was not traumatic, avoided complications and had the best outcome for the patient that was satisfying: For instance:

*I think I just love needling; I take a lot of pride[in] my needling. I am devastated when I miss. (Emma)*

Being able to assist the patient towards a better quality of life and promoting self-care were described as a feeling of success. For instance, Zoe described that:

*Knowing you’ve done all you could possible do to make the patient’s life better for that day and to improve their quality of life. (Zoe)*

Nurses commented on the ability to make autonomous decisions about aspects of patient care. Nurses working in haemodialysis are known for their specialist knowledge and often work with limited medical input. This affords nurses an enhanced scope of practice and leads to autonomous decision-making. Susan described her sense of autonomy as:

*You might run something past a doctor but pretty much the decision making is very nursing based – a nurse practitioner type environment and I think it’s one of the areas in renal that we are sort of almost already doing that* [nurse] *practitioner role without an official title. (Susan)*

#### Intense working teams

The final theme describes the interactions that nurses have with their nursing colleagues which was viewed along a continuum from supportive to stressful interactions. The intense working team was due to the feeling of comradeship from being within a close knit group of colleagues who provided support, mutual encouragement and respect. For instance Emma explained how her colleagues:

*Supporting each other; not bitching. I think that the most important thing is just basically supporting each other and recognizing that everybody has a bad day, and then in our unit particularly which I think is a really good thing – everybody gets in and helps everybody, and I think that’s where it is really a happy place at the moment. (Emma)*

Interactions between colleagues were also perceived as a source of stress with triggers being patient care decisions and when nurses felt a lack of support within their workplace. Haemodialysis nursing teams are typically small with dialysis units frequently isolated from other hospital wards or located off hospital campuses. Working within small teams can make it difficult to find support in times of need due to lack of confidentiality and empathy from colleagues.

*I know a lot of the units too they are quite isolated, so there might only be 1 or 2 staff on so they’re it. And when you are constantly coping with their issues – without having enough staff to bounce off; without having the support of somebody else to say hey what’s happening here. (Emma)*

A healthy team environment was important for haemodialysis nurses when faced with intense workloads where patient load, resources and staffing are repeatedly stretched. Workload intensity although described by all nurses who were interviewed was particularly problematic for those nurses who worked in the in-center haemodialysis units where patients have higher acuity levels requiring more frequent and complex physical and psychological interventions. The nurses described heavier and unrealistic workloads as a source of potential burnout which was accentuated by stressful relationships within the team. Elizabeth for example stated that:

*I think from where I’ve worked burnout would have a lot to do with unexpected patients coming and having to squeeze them in say if you’ve got six chairs and 8 people and you’re trying to push one in and push one out and the units I’ve worked in are pretty much like a cauldron, it’s very confined and the intensity if there’s stress in there…it’s just like a fireball and nowhere to go and cool off even just to walk away…it’s often very intense (Elizabeth)*

The importance of a cohesive supportive team and its relationship with burnout was emphasized by Emma who stated that

*If you don’t have a supportive team within your unit, then I think burnout could happen quite regularly. (Emma)*

### Integration of quantitative and qualitative findings

Due to the nature of the sequential explanatory research design the findings from the qualitative phase supported the quantitative findings but also provide contextual meaning. Flexible management was recognized by nurses in the quantitative phase as being the most important factor with a satisfying work environment. The subscale of flexible management focuses on the tasks that a manager does such as preparing the roster and helping in times of need. The tasks that the nurse unit manager did were not, however, identified during interviews instead managers were seen as a support person and as someone who helped make the haemodialysis unit a happy place to work. The nurses who participated in the interviews described the nurse manager’s role in ensuring that there was adequate staffing which influenced their ability to care and the workload.

The satisfying interactions that a nurse had with others (patients, nurses, other healthcare professionals) in the workplace were described during the interviews as being juxtaposed with being a source of stress. Nurses identified that working in a team atmosphere was satisfying but also the lack of support that they received from colleagues at crucial times caused stress. This finding might explain why interactions with nursing colleagues did not score highly in the quantitative phase. In addition the interactions that a nurse had with patients was not measured during the quantitative phase so the qualitative findings, where nurses described not only the reward of having a long-term relationship with patients but also the stress that occurs when a patient dies, adds valuable insight into the factors contributing to job satisfaction and stress amongst haemodialysis nurses. The Index of Work Satisfaction measures nurse/doctor interactions and in this sample, these interactions were found satisfying, however the themes ‘patients as quasi-family’ and ‘intense working teams’ focus on different relationships. Both of these themes explained that for haemodialysis nurses, interactions with patients and other nurses contribute to job satisfaction as well as job stress.

The nurses interviewed reported that a satisfying aspect of the job was the ‘ability to care’ and this reflects that the nurses’ focus was patient centered with the goal to cause minimal discomfort or complications. The feeling of providing good nursing care was another area where the instruments did not capture the significance of the intrinsic rewards that nurses received from performing their job in the haemodialysis unit.

Workloads were identified in both the quantitative and qualitative phases as causing stress. Workload was the highest stressor in the Nursing Stress Scale due to having insufficient time to care, inadequate breaks, and poor levels of staffing. The theme ‘intense working teams’ captures the stressful working environment and provided context and explanation for the high scores found in the Nursing Stress Scale. Nurses commented in the interviews that when there was a heavy workload, care became task orientated (i.e. technical aspects of the delivery of dialysis treatment) leading to the perception of providing lower quality care. The focus on tasks meant that nurses had an inability to care and this led to decreased job satisfaction.

## Discussion

This study used a sequential explanatory mixed methods design to explain haemodialysis nurses experience of satisfaction with the work environment, job satisfaction, job stress and burnout.

High levels of both job satisfaction and emotional exhaustion were found [9], and that the high levels of emotional exhaustion were linked to stress of the job rather than due to decreased job satisfaction [39]. These results highlight that the job that haemodialysis nurses perform provides satisfaction but it comes at a cost to their emotional wellbeing leading to burnout. In addition, the context of prolonged relationships with patients in the haemodialysis environment contributes to job satisfaction but that over time this relationship becomes emotionally draining and a source of stress particularly when patients die. Traditionally nurses are encouraged to provide a stoic persona in the face of emotional pressures in the workplace [67], however, objective stoicism is difficult when close personal relationships are formed with patients.

This study also highlights that nurses in the haemodialysis environment need to be aware of the stressors that exist in their workplace. Increased support from nurse managers and colleagues can act as a buffer against the stressors of workloads, the intense personal relationship, and repeated exposure of patient death [68–70]. To be responsive to the emotional cost of the job, nurse managers need to actively facilitate psychological support of nurses. This could be achieved through formal programs to promote psychological wellbeing and team cohesiveness. Strategies may include mentoring, formal collegial support and increased nurse manager support [71]. Regular performance reviews that specifically include measurement of stress levels and a discussion on appropriate coping strategies could also assist haemodialysis nurses to prevent or to institute timely interventions to avoid the development of burnout [72].

For job satisfaction the intrinsic rewards described during interviews reinforced the quantitative results. Nurses felt a sense of intrinsic reward from being autonomous in their practice and by being technically proficient in the skills they were required to perform. Intrinsic reward, according to Herzberg, Mausner, and Snyderman [73] is a feeling of self-actualization or self-accomplishment. In this study nurses gained intrinsic rewards through nurse-led, patient centered decision-making. Nurses reported being able to make these autonomous decisions based on their specialized skills and knowledge (e.g. deciding on interventions during dialysis complications). Autonomy is a core component of job satisfaction [74] and is “the amount of job-related independence, initiative, and freedom either permitted or required in daily work activities p.60” [55]. The specialized nature of haemodialysis allows nurses who have gained expertise the ability to be autonomous in deciding care plans for patients [3], and also reflects the limited input medical staff have in the provision of delivering the haemodialysis treatment. Limited input by medical staff also allowed for haemodialysis nurses to have a wider scope of practice than is typically found in other areas of nursing [75]. Development of formal advanced nursing practice roles (e.g., nurse practitioner and clinical nurse consultant/specialist) would facilitate greater autonomy and decision making, and could allow nurses to gain greater job satisfaction. Advanced practice roles offer the ability to have a wider scope of practice and have demonstrated higher levels of job satisfaction and improved patient outcomes [76].

Being able to provide good care through technical proficiency was another area where nurses derived feelings of success. The haemodialysis environment is a highly technical area where nurses are required to develop complex skills not found in other areas of nursing. Nurses spoke of a sense of pride in being able to perform activities such as comprehensive physical assessment of patients to determine dialysis prescription requirements, cannulating arteriovenous fistulae to achieve maximal blood flows for optimally efficient dialysis (e.g., 400 ml/min), anticipating potential life-threating patient complications (e.g. severe hypotension), and being able to troubleshoot haemodialysis machine problems in a proficient manner. Through these activities they gained a sense a pride in their skills and professional status among their peers. The concept of technical proficiency was a contributor to haemodialysis nurses’ job satisfaction that was not measured in the Index of Work Satisfaction so identifying other types of intrinsic rewards may be helpful in understanding job satisfaction in other highly technical nursing environments.

Professional interactions were identified during the quantitative phase as being important to job satisfaction [9]. During the qualitative phase this was noted especially in the supportive relationships with other nurses and led to a sense of team work and unity. Nurses commented on how team cohesiveness enhanced job satisfaction and provided a support network for nurses in times of increased stress. This result is consistent with Brown et al. [4] who identified haemodialysis work to be emotionally taxing but that nurses do not realize the emotional labor involved with the job and are not always certain of their co-workers’ support. A team atmosphere in the work place has been demonstrated to reduce emotional labor and stress leading to less burnout and greater retention of registered nurses [77]. The development and sustaining of effective teams in dialysis units could reduce the stress of coping with the type of work experienced by these nurses.

An area that has not been reported previously is the impact of the intense relationship that is formed between the nurse and patient in the haemodialysis environment. The frequent, intense and prolonged interaction led to blurring of the traditional therapeutic relationship and the development of a quasi-family for the nurse and the patient. Nurses reported having intense grief when a patient died whom they had forged a close relationship with. Grief has been identified in other areas of nursing where nurses experience emotional distress, compassion fatigue and staff turnover as a result of caring for patients who were dying [78]. The concept of compassion fatigue amongst haemodialysis nurses warrants further investigation given the high score on the Nursing Stress Scale in this study and the impact of patients dying on the emotional wellbeing of nurses that was frequently described during interviews.

Through the use of mixed-methods research, this study has identified that there are some facets of haemodialysis nursing that fall outside the boundaries of validated instruments. This raises the possibility that as nursing becomes more specialized that previous instruments may not be capturing the complexity of the different work environments, aspects that nurses find satisfying or stressful, or those contributing to burnout. Further research could lead to the development of a haemodialysis nurse specific instrument that better measures factors identified in this study such as the impact of the nurse-patient relationship.

### Limitations

The strength of this study is the large sample obtained during the quantitative phase from two countries with similar haemodialysis practices making it one of the largest studies undertaken globally in this population. Even though the qualitative phase consisted of eight interviews, the technique of maximum variation sampling lead to a range of participants’ perspectives being sought. There was however a limitation of this study in that participants were drawn from a voluntary professional nursing organization which may comprise more motivated and committed nurses compared to non-members that may limit the generalizability of the results.

## Conclusion

Through the use of a sequential explanatory mixed methods design, a two phased approach to explore haemodialysis nurses current levels and experience of satisfaction with the work environment, job satisfaction, stress and burnout was undertaken. Overall nurses were satisfied with their work environment and being able to care for patients with complex healthcare needs but there were stressors that lead to increased emotional strain. The haemodialysis stressors were the intense relationship between the nurse and patients and the impact of recurrent grief.

This study provides valuable insight into the nature and complexity of work as a haemodialysis nurse and will enable nurses, nurse managers and organizations to better understand the rewards and stresses that these nurses face. The leadership role of nurse managers in stress management is crucial in establishing and sustaining a work environment conducive to job satisfaction and increased workforce retention.
